# Differences in perinatal outcomes in teenage mothers with their first and third pregnancies and predictors of adverse neonatal events: A cross-sectional study

**DOI:** 10.18502/ijrm.v19i11.9916

**Published:** 2021-12-13

**Authors:** Shaymaa Kadhim Jasim, Hayder Al-Momen, Ali Abdul Razzak Obaid

**Affiliations:** ^1^Department of Obstetrics and Gynecology, College of Medicine, University of Baghdad, Baghdad, Iraq.; ^2^Department of Pediatrics, Al-Kindy College of Medicine, University of Baghdad, Baghdad, Iraq.

**Keywords:** Teenage pregnancy, Complications, Neonate.

## Abstract

**Background:**

Repeated teenage pregnancy is a major burden on the healthcare system worldwide.

**Objective:**

We aimed to compare teenagers with their first and third pregnancies and to evaluate the likelihood of neonatal complications.

**Materials and Methods:**

This cross-sectional study was performed on female teenagers (aged ≤ 19 yr) with singleton pregnancies. The subjects (n = 298) were screened over 12 months. Ninety-six women were excluded, based on the exclusion criteria. The remaining subjects (n = 202) were divided into two groups: teenagers with first pregnancy (n = 96) and teenagers with third pregnancy (n = 47). The subjects were observed throughout pregnancy and delivery. The final sample size of the first and third pregnancy groups was 96 and 47, respectively.

**Results:**

There was a significant risk of preeclampsia in the first pregnancy group (p = 0.01). Low birth weight, five-min Apgar score < 7, and neonatal intensive care unit admission were the most significant neonatal outcomes in the first pregnancy group. In the third pregnancy group, significant predictors of neonatal complications included very young age in the first pregnancy ( ≤ 15 yr), an inter-pregnancy interval < 2 yr, current anemia, and history of obstetric and/or neonatal complications in previous pregnancies.

**Conclusion:**

Based on the results, teenagers with their first pregnancy had comparable obstetric outcomes (except for preeclampsia) as teenagers with their third pregnancy, whereas neonatal complications occurred more frequently in the first pregnancy group. Overall, we can predict high-risk neonates in the third pregnancy, based on the abovementioned parameters.

## 1. Introduction

Teenage pregnancy occurs when women aged ≤ 19 yr become pregnant. The frequency of this phenomenon has increased in different parts of the world and is no longer limited to developing countries, as high rates have also been reported in developed countries, including the United States (1, 2). Teenage pregnancy has been discussed in many previous studies, and its causes and complications have been investigated as major issues in health policymaking and programs throughout the world (3, 4).

The impact of teenage pregnancy is variable in different communities. In developed countries, lack of common sexual health education programs and contraceptive measures has contributed to this phenomenon, which occurs mostly outside marriage (5). Nonetheless, these countries have a better status than developing countries in terms of teenage pregnancy. This may be due to the fact that in developing countries, the public accepts the idea of teenage marriage because of the cultural background and traditions (5, 6). At a social level, teenage pregnancy imposes significant pressure on new couples with poor academic performance and a low socioeconomic status.

Moreover, many adverse events may occur during or after gestation, such as anemia, preeclampsia, obstructed labor, preterm delivery, postpartum depression, and even maternal death, which has been shown to be five times more likely than in older women (7, 8). The newborns are also more likely to be exposed to several complications, such as prematurity and low birth weight (LBW), which can have acute and remote effects on the newborn and family. Almost 15% of delivered newborns in the Persian Gulf area of the Middle East are born to teenage mothers. However, in Iraq, a country with a similar culture and belief system to other Persian Gulf countries, there is no precise estimation or evidence regarding pregnancy during adolescence (6, 9, 10).

Since in Iraq, adolescents may marry and conceive at a very young age (starting from 11 yr), and it is socially advised to have multiple pregnancies within short intervals (6, 11), in this study, we aimed to evaluate and compare perinatal outcomes between teenagers with their first and third pregnancies, and to investigate the risk factors for adverse neonatal events in the third pregnancy. According to our literature search, no such comparisons have been made in published studies. Therefore, the present findings might help us have a better understanding of obstetric and neonatal outcomes in teenage pregnancies and identify the predictors of neonatal complications in third teenage pregnancies.

## 2. Materials and Methods

In this prospective, cross-sectional study, all women aged ≤ 19 yr with singleton pregnancies, who started their first antenatal care (ANC) visit during their first trimester at the Obstetrics and Gynecology Department of Medical City Hospital in Baghdad, Iraq, were considered eligible for the study and recruited from August 2018 to the end of July 2019. It should be noted that our hospital is a large tertiary care center, treating patients from all around the country.

Pregnancy was confirmed when human chorionic gonadotropin levels > 25 mIU/mL were detected in blood tests. The inclusion criteria were as follows: (1) pregnant teenage women (aged ≤ 19 yr) with their first or third pregnancy; (2) presentation to hospital for the first ANC visit during the first trimester at or before six to seven wk of gestation; (3) singleton pregnancy; (4) complete follow-up with regular ANC visits; and (5) absence of systemic or medical problems during the study.

The exclusion criteria were as follows: (1) presence of chronic diseases in the first presentation, such as cardiac, renal, or thyroid disorders, diabetes mellitus, hypertension, or hematological disorders; (2) multiple pregnancy; and (3) delivery outside our hospital or failure to contact the researcher during pregnancy at least by phone call.

**Figure 1:**
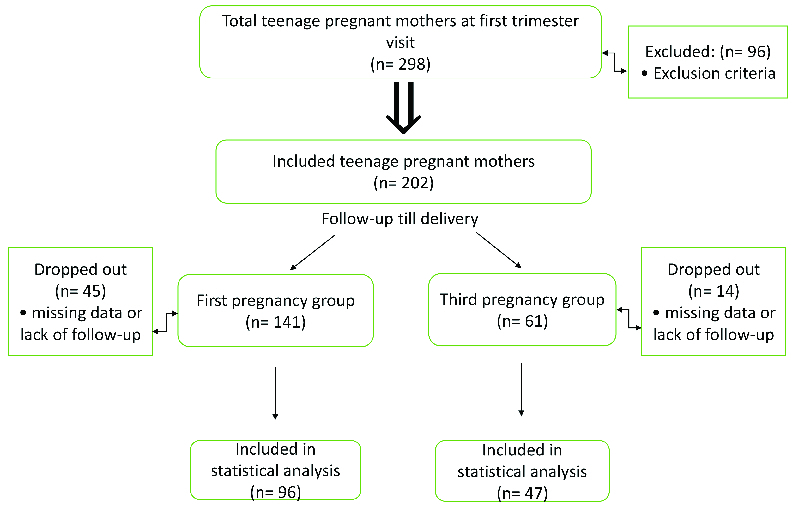
Flowchart of sample recruitment.

According to the inclusion criteria, the total number of screened pregnant teenagers was 298, 96 of whom were excluded, based on the exclusion criteria. The remaining women (n = 202) were categorized into two groups in terms of gravidity. The first group included women with their first pregnancy (n = 141), while the second group included women with their third pregnancy (n = 61). However, in the first and third pregnancy groups, 45 and 14 women were excluded, respectively, due to missing data (delivery outside our hospital), lack of regular follow-up visits, or lack of contact with the researchers. The net sample of women was 96 in the first pregnancy group and 47 in the third pregnancy group (Figure 1).

Both groups had an equal number of ANC visits and were followed up throughout gestation. Full medical history-taking, examinations, and imaging studies (ultrasonography) were performed by the attending obstetrician, in addition to primary routine assessments, based on the hospital's guidelines, including blood sugar, hemoglobin and urinary protein tests. After delivery, a neonatologist cared for the neonates with a complete workup profile, as needed.

### Ethical considerations

Ethical approval was granted by the Ethics and Scientific Committee of the College of Medicine and Al-Kindy College of Medicine (No: 837), affiliated with the University of Baghdad. Informed consent was also obtained from all participants. We strictly adhered to the Declaration of Helsinki guidelines in this study.

### Statistical analysis

Data were evaluated in the Statistical Package for the Social Sciences version 22.0 for Windows (SPSS Inc., Chicago, IL, USA). An independent *t* test was used for evaluating the continuous variables, and Fisher's exact test was used for the categorical variables. A logistic regression model was used to analyze the potential risk factors for neonatal complications in the third pregnancy by measuring the odds ratios and 95% confidence intervals. The level of statistical significance was set at p < 0.05.

## 3. Results

The rate of third pregnancy in our sample of teenage mothers was 22.93% (61/266), while the rate of first pregnancy was 53.00% (141/266). The first and third pregnancy groups consisted of 141 and 61 women, respectively. However, only 96 and 47 women were included, respectively, as 45 and 14 women were excluded, respectively, because of missing data or lack of regular follow-ups (Figure 1).

### General information of the groups

Both groups of pregnant teenagers with their first or third pregnancies (96 and 47, respectively) had comparable sociodemographic characteristics, as shown in table I. Also, table II shows that only preeclampsia occurred more frequently in the first pregnancy group as compared to the third pregnancy group (p = 0.01). Maternal anemia had a high prevalence in both groups (higher in the first pregnancy group), without any significant difference between the groups. Other parameters showed no significant differences between the groups.

### Neonatal complications 

Neonatal outcomes, including LBW, five-min Apgar score < 7, and neonatal intensive care unit (NICU) admission, were significantly more common in the first pregnancy group, as shown in table III.

### Predictors of neonatal outcomes

The odds ratios and 95% confidence intervals were calculated by performing a logistic regression analysis to identify the significance of possible etiological factors in influencing neonatal complications of the third pregnancy group (Table IV). The educational level and socioeconomic status of teenage mothers were adjusted for in our analysis. The significant risk factors for neonatal complications in the third pregnancy group included a short interval between the second and third pregnancies ( < 2 yr); maternal age < 15 yr at first gestation; a history of obstetric and/or neonatal adverse events in previous pregnancies; and a diagnosis of maternal anemia in the third pregnancy. However, weight gain during the third pregnancy showed no significant association.

**Table 1:** General characteristics of teenage pregnant women


** Maternal characteristics**		**First pregnancy group**	**Third pregnancy group**	**p-value**
Age (yr)*		15.60 ± 2.34	17.10 ± 1.87	0.13
BMI at presentation (kg/m ${}^{2}$ )*		26.4 ± 3.6	26.9 ± 4.2	0.29
Gestational age (wk) at delivery*		36.9 ± 3.1	37.6 ± 3.4	0.21
Consanguinity**		34 (35.42)	16 (34.04)	0.28
Place of residence**				
	**Rural**	63 (65.63)	33 (70.21)	
	**Urban **	33 (34.37)	14 (29.79)	0.19
Smoking**		3 (3.13)	1 (2.13)		0.56
Educational level**				
	**Literate**	73 (76.04)	37 (78.72)	
	**Illiterate**	23 (23.96)	10 (21.28)	0.71
Occupational status**				
	**Employed**	15 (15.62)	6 (12.77)	
	**Housewife**	81 (84.38)	41 (87.23)	0.43
*Data are presented as Mean ± SD (Student's *t* test), **Data are presented as n (%) (Fisher's exact test), BMI: Body mass index				

**Table 2:** Obstetric outcomes in the first and third pregnancy groups


** Adverse events**		**First pregnancy group (n = 96)**	**Third pregnancy group (n = 47)**	**p-value**
Anemia*		85 (88.54)	38 (80.85)	0.27
Gestational weight gain (kg)**		10.9 ± 5.9	10.6 ± 5.4	0.39
Preeclampsia*		17 (17.71)	4 (8.51)	0.01
Premature rupture of membranes*		5 (5.21)	2 (4.26)	0.74
Gestational diabetes*		2 (2.08)	1 (2.13)	1.00
Polyhydramnios*		4 (4.17)	2 (4.26)	1.00
Oligohydramnios with no ruptured membranes*		5 (5.21)	2 (4.26)	0.74
Postpartum hemorrhage*		3 (3.13)	1 (2.13)	0.69
Delivery mode*				
	**Normal vaginal delivery (spontaneous)**	49 (51.04)	22 (46.81)	
	**CS**	47 (48.96)	25 (53.19)	0.43
*Data are presented as n (%) (Fisher's exact test), **Data are presented as Mean ± SD (Student's *t *test), CS: Cesarean section				

**Table 3:** Neonatal complications in the pregnancy groups


**Complications**	**First pregnancy group**	**Third pregnancy group**	**p-value**
Preterm neonates*	26 (27.08)	9 (19.15)	0.45
Birth weight (kg)**	2352 ± 429	2586 ± 417	0.06
LBW (1.5-2.5 kg)*	29 (30.21)	6 (12.77)	0.01
VLBW ( < 1.5 kg)*	5 (5.21)	2 (4.26)	0.74
Congenital malformation*	3 (3.13)	1 (2.13)	0.69
Five-min Apgar score < 7*	11 (11.45)	3 (6.38)	0.02
NICU admission*	22 (22.92)	5 (10.63)	0.01
Stillbirth and early neonatal death*	6 (6.25)	2 (4.26)	0.53
*Data are presented as n (%) (Fisher's exact test), **Data are presented as Mean ± SD (Student's *t* test), LBW: Low birth weight, VLBW: Very low birth weight, NICU: Neonatal intensive care unit			

**Table 4:** Neonatal complications and potential risk factors in the third pregnancy group


	**Complications**		
**Risks**	**Presence**	**Absence**	**OR (95% CI)**	**p-value**
Inter-pregnancy interval < 2 yr*	5 (45.45)	10 (27.78)	1.5 (0.3-1.8)	0.03
Maternal age at first pregnancy ≤ 15 yr**	13.2 ± 1.78	17.5 ± 1.12	1.7 (0.8-2.3)	0.02
Obstetric complications in previous pregnancies*	6 (54.55)	12 (33.33)	1.8 (0.9-3.2)	0.01
Neonatal complications in previous pregnancies*	5 (45.45)	11 (30.55)	1.3 (0.9-1.8)	0.02
Current maternal anemia*	10 (90.91)	28 (77.78)	1.6 (0.5-1.8)	0.03
Weight gain in the third pregnancy**	11.9 ± 2.1	11.7 ± 3.6	0.7 (0.4-1.2)	0.16
*Data are presented as n (%) (Fisher's exact test), **Data are presented as Mean ± SD (Student's *t* test), OR (95% CI): Odds ratio (95% confidence interval)				

## 4. Discussion

While teenage pregnancy is a common problem in developing countries, it is also not uncommon in developed regions. This phenomenon imposes a significant burden on the healthcare systems of all countries due to its associated complications during pregnancy and after delivery. Therefore, major attempts have been made for better identification and management of these complications (12, 13). To the best of our knowledge, this is the first study to discuss these adverse events in teenagers with three pregnancies during adolescence.

In the present study, teenagers with their first and third pregnancies had comparable sociodemographic characteristics, unlike a previous Japanese study, which compared junior teenagers ( < 16 yr) with senior teenagers (16-19 yr), regardless of parity or gravidity, and found that younger teenagers were thinner and shorter and had more smoking habits (7). In our study, the first pregnancy group, which was the younger age group, had a lower mean weight and a higher smoking rate, compared to the older group with the third pregnancy, although these differences were not significant; these findings could be partly in agreement with the Japanese study results.

Maternal anemia was slightly more prevalent in our first pregnancy group, although the difference was not significant. Despite the high rate of maternal anemia in both groups, it was close to the local maternal anemia estimates (14). Overall, increased rates of anemia have been reported in teenage pregnant women as compared to older adults. According to some studies, the younger a teenage pregnant woman is, the lower her hemoglobin level will be (15, 16). In the present study, lower rates of delivery by cesarean section (CS) were observed in the first pregnancy group, which is in line with previous studies, suggesting the decreased frequency of CS in teenage pregnant women (17, 18). Also, the rates of CS delivery were lower in both groups as compared to the national Iraqi CS rates for all age groups (19). On the other hand, Turkish researchers documented higher rates of CS in teenagers due to the immaturity of pelvic organs, leading to possible disproportions (8); this discrepancy between the results might be related to racial differences.

The frequency of preeclampsia was significantly higher in the first pregnancy group as compared to the third pregnancy group (p = 0.01). Many previous studies have also suggested that preeclampsia, eclampsia, and pregnancy-induced hypertension occur more frequently among teenagers (17, 18, 20, 21). On the other hand, other studies from Oman and Canada found no significant differences in preeclampsia or obstetric complications between teenage and adult mothers (16, 22). Such differences might be attributed to the size of samples in different studies, biological risks, and social factors.

The present results showed that other obstetric parameters, such as prelabor rupture of membranes and postpartum hemorrhage, were analogous in the first and third pregnancy groups, without any significant difference. This finding was also reported in a study from the United States on young and older teenage pregnant women, besides other local studies evaluating maternal complications in different age groups (6, 20). We found that the first pregnancy group had more neonatal complications compared to the third pregnancy group, including LBW, five-min Apgar score < 7, and NICU admission. Other adverse events in newborns were similar between the groups, such as preterm delivery, very low birth weight, congenital abnormalities, stillbirth, and early neonatal death. This finding is consistent with many previous reports that have confirmed the high level of neonatal complications in teenagers (23-25). The high incidence of neonatal complications could be explained in part by the deficiency of health education for teenage pregnant women and their lack of interest in or fear of pregnancy (22-24).

Some researchers have found higher rates of congenital anomalies in teenage pregnant mothers, while others have revealed no significant difference in the rate of poor neonatal outcomes between very young (13-16 yr) and young (17-19 yr) teenage mothers, regardless of parity (26, 27). The discrepancy between previous studies could be explained by different factors that are assumed to be linked to poor neonatal outcomes, such as very young maternal age, low income, poor socioeconomic status, food insecurity, alcohol use, smoking, and substance abuse (18, 27-29).

In the present study, potential confounders that could affect neonatal complications were controlled for. These confounders included biological, demographic, and socioeconomic characteristics, including maternal weight, gestational age at delivery, consanguinity, regular ANC, place of residence, smoking, alcohol consumption, employment status, income, and food insecurity (low availability or quality). In this study, neonatal complications occurred more frequently in adolescents with their third pregnancy if they were very young ( ≤ 15 yr); had an inter-pregnancy interval of < 2 yr; experienced anemia in the current pregnancy; or had a history of obstetric and/or neonatal complications in previous pregnancies.

The mentioned findings of the present study are in line with reports from different countries, showing that younger teenage mothers had a higher risk of adverse pregnancy and neonatal outcomes (20, 27). Conversely, a Zambian study reported that maternal age had an insignificant association with gestational and neonatal complications (30). However, this retrospective study only included samples from a limited area in Zambia, which might have affected the results. In line with our results, a study from Ethiopia found preeclampsia to be the main obstetric complication, leading to LBW, and considered it as a major neonatal complication (10).

In this study, repeated teenage pregnancy with short intervals was an important predictor of adverse neonatal outcomes. A previous study found that an inter-pregnancy interval of less than 18 months could have some consequences for teenage mothers. Also, a previously published paper and a recent systematic review revealed that neonatal birth was influenced by repeated teenage pregnancy (31, 32). Also, poor educational and socioeconomic status of teenage mothers had major contributions to the high rate of maternal anemia, as these mothers underestimated the importance of hemoglobin measurements and regular ANC visits; even when they participated in such measurements, iron and folic acid supplementations were used irregularly with poor compliance. However, the mentioned risk factors for maternal anemia were adjusted for in our analysis, and maternal anemia had a direct impact on neonatal well-being, especially birth weight, as reported in other studies (33, 34).

### Strengths and limitations

The sample of our study was selected from one setting. Although our hospital is the largest obstetric center in the country, it is strongly recommended that future studies include more hospitals to have a larger sample size. A strength of the study was that, by using a prospective approach, we could monitor our teenage mothers and investigate differences between the first and third pregnancy groups, which have never been discussed in previous studies. Finally, we could not find any other studies measuring obstetric and neonatal complications in teenagers with third pregnancies, despite the high rates of multiparity in these mothers throughout the world, so this can also be considered a strength of our study. About 22.5 million teenage girls become pregnant in 60 underdeveloped countries in 2017, of whom 4.1 million teenagers had multiple pregnancies ( ≥ 2 pregnancies). Also, in Europe, similar figures have been reported. For example, in 2018, the rate of third pregnancy in Romania was 6.1% out of a total teenage pregnancy rate of 11.1%; in other words, more than half of teenage mothers tended to have a third pregnancy, which is much higher than the rate measured in our study (22.93%). Also, the corresponding rate in the United States was estimated at 20% for multiple pregnancies in teenagers in 2015 (27, 32, 35).

## 5. Conclusion

Teenage mothers with their first pregnancy had comparable obstetric complications as teenage mothers with their third pregnancy, except for preeclampsia, which was significantly more likely in the first pregnancy group. Regarding the neonatal outcomes, LBW, five-min Apgar sore < 7, and NICU admission were significantly more common in the first pregnancy group. Other neonatal outcomes were also more frequent in the first pregnancy group as compared to the third pregnancy group, although these differences were not significant. Maternal age ≤ 15 yr, rapid repeated pregnancies (inter-pregnancy interval < 2 yr), maternal anemia in the current pregnancy, and the presence of obstetric and/or neonatal complications in previous pregnancies were possible predictors of future neonatal adverse events in the third pregnancy.

##  Conflict of Interest

The authors declare no conflict of interest.
